# A multivariate model for successful publication of intensive care medicine randomized controlled trials in the highest impact factor journals: the SCOTI score

**DOI:** 10.1186/s13613-021-00954-x

**Published:** 2021-11-27

**Authors:** Joris Pensier, Audrey De Jong, Gerald Chanques, Emmanuel Futier, Elie Azoulay, Nicolas Molinari, Samir Jaber

**Affiliations:** 1 Department of Anesthesia and Intensive Care Unit, Regional University Hospital of Montpellier, St-Eloi Hospital, University of Montpellier, PhyMedExp, INSERM U1046, CNRS UMR, 9214 CEDEX 5 Montpellier, France; 2 Department of Peri-Operative Medicine, CHU Clermont-Ferrand, 63000 Clermont-Ferrand, France; 3 Médecine Intensive et Réanimation, Groupe FAMIREA, Hôpital Saint-Louis, Université de Paris, Paris, France; 4 IDESP, INSERM, Univ Montpellier, CHU Montpellier, Montpellier, France; 5 Universite de Montpellier, Montpellier, Languedoc-Roussillon, France; 6 Département d’Anesthésie Réanimation B (DAR B), 80 Avenue Augustin Fliche, 34295 Montpellier, France

**Keywords:** Intensive care unit, Critical care, Critically ill patients, Intensive care medicine, Precision medicine

## Abstract

**Background:**

Critical care randomized controlled trials (RCTs) are often published in high-impact journals, whether general journals [the New England Journal of Medicine (NEJM), The Lancet, the Journal of the American Medical Association (JAMA)] or critical care journals [Intensive Care Medicine (ICM), the American Journal of Respiratory and Critical Care Medicine (AJRCCM), Critical Care Medicine (CCM)]. As rejection occurs in up to 97% of cases, it might be appropriate to assess pre-submission probability of being published. The objective of this study was to develop and internally validate a simplified score predicting whether an ongoing trial stands a chance of being published in high-impact general journals.

**Methods:**

A cohort of critical care RCTs published between 1999 and 2018 in the three highest impact medical journals (NEJM, The Lancet, JAMA) or the three highest impact critical care journals (ICM, AJRCCM, CCM) was split into two samples (derivation cohort, validation cohort) to develop and internally validate the simplified score. Primary outcome was journal of publication assessed as high-impact general journal (NEJM, The Lancet, JAMA) or critical care journal (ICM, AJRCCM, CCM).

**Results:**

A total of 968 critical care RCTs were included in the predictive cohort and split into a derivation cohort (*n* = 510) and a validation cohort (*n* = 458). In the derivation cohort, the sample size (*P* value < 0.001), the number of centers involved (*P* value = 0.01), mortality as primary outcome (*P* value = 0.002) or a composite item including mortality as primary outcome (*P* value = 0.004), and topic [ventilation (*P* value < 0.001) or miscellaneous (*P* value < 0.001)] were independent factors predictive of publication in high-impact general journals, compared to high-impact critical care journals. The SCOTI score (Sample size, Centers, Outcome, Topic, and International score) was developed with an area under the ROC curve of 0.84 (95% Confidence Interval, 0.80–0.88) in validation by split sample.

**Conclusions:**

The SCOTI score, developed and validated by split sample, accurately predicts the chances of a critical care RCT being published in high-impact general journals, compared to high-impact critical care journals.

**Supplementary Information:**

The online version contains supplementary material available at 10.1186/s13613-021-00954-x.

## Background

Since the late twentieth century, evidence-based medicine has emerged as a new paradigm to produce knowledge and improve clinical practice [1]. Randomized controlled trials (RCTs) are considered as the pinnacle of evidence for evaluating new and existing interventions in medicine [2], as long as they are appropriately designed, conducted, and reported [3, 4]. They have been further promoted by the development of online databases, reviews, meta-analyses, and education of clinicians and students [5].

Critical care is no exception. Many RCTs have been performed in this field and have often led to publications in the highest impact critical care journals, such as Intensive Care Medicine (ICM), the American Journal of Respiratory and Critical Care Medicine (AJRCCM), and Critical Care Medicine (CCM) [6–8]. Moreover, landmark trials have changed the management of critically ill patients [9–12]. Many of those studies have been published in the highest impact general journals: The New England Journal of Medicine (NEJM), The Lancet, and the Journal of the American Medical Association (JAMA).

It is the final purpose of medical research to broadcast its results as widely as possible to health care practitioners to most impact the management of patients worldwide [13]. In this context, RCTs performed in the field of critical care aim to be published in the highest impact general journals [2]. As up to 97% of trials are rejected outright or post-review, it might be appropriate to assess pre-submission probability of being published. To our knowledge, no study has yet explored the factors associated with the publication of a critical care RCT in a high-impact general journal.

We designed this cohort of studies to identify independent predictive factors of publication of a critical care RCT in a high-impact general journal (NEJM, Lancet, JAMA) compared to the high-impact critical care journals (ICM, AJRCCM, CCM). The secondary objectives of this study were to develop and validate a simplified score predicting publication of an RCT in a high-impact general journal. We hypothesized that most predictive factors of publication of a critical care RCT are determined before the inclusion of its first patient.

## Materials and methods

### Study design

We conducted a retrospective cohort of RCTs according to the Transparent Reporting of a Multivariable Prediction Model for Individual Prognosis or Diagnosis (TRIPOD) reporting guideline statement [14]. The cohort of RCTs was gathered as a systematic review of RCTs performed in critically ill patients between 1999 and 2018, following the Preferred Reporting Items for Systematic reviews and Meta-Analyses (PRISMA) statement [15].

### Data sources and study selection

The search strategy is detailed in the electronic supplementary material (Additional file 1: eAppendix S1). We screened for relevant RCTs performed: (1) on critically ill patients, (2) that enrolled adults, and (3) were published between 1999 and 2018, (4) in the three highest impact general journals [The New England Journal of Medicine (NEJM), The Lancet and the Journal of the American Medical Association (JAMA)] or the three highest impact critical care journals [Intensive Care Medicine (ICM), American Journal of Respiratory and Critical Care Medicine (AJRCCM) and Critical Care Medicine (CCM)]. RCTs performed on animals, on pediatric patients, on non-critically ill patients, bench studies, and simulation studies were not eligible. The three journals for each category (general journals and critical care journals) were selected according to the values of their mean Impact Factor obtained over 3 years from 2016 to 2018 [16]. We chose to only take into account the three “princeps” general journals, which were continuously available from 1999 to 2018. The “sister” journals of a general journal (such as Lancet Respiratory Medicine or JAMA Network) were not included in our research, as they have not been continuously available over the 20-year period, and their respective impact factors are not in the same range as the “princeps” journals.

### Data collection

First, two authors (JP and ADJ) independently screened the studies retrieved by title and then by abstract for exclusion. They assessed the full text of possibly relevant studies for inclusion and exclusion criteria. Disagreement was resolved by discussion and arbitrated, if necessary, by a third author (SJ). Data were then added to an excel database, specifically designed for this review [15]. Journal of publication, year of publication, sample size, numbers of centers involved, country of the first author, number of countries participating, primary endpoint of the RCT, the result of the RCT according to its primary endpoint, the type of intervention tested, and the topic of the RCT were extracted.

Journal of publication was classified either as a high-impact general journal (NEJM, Lancet, JAMA) or as a high-impact critical care journal (ICM, AJRCCM, CCM).

The result of the RCT was classified either as unsignificant, significant for benefit, or significant for harm. We adapted a previously published classification [17] to include equivalence and non-inferiority designs. It was considered unsignificant if the *P* value was higher than 0.05 for superiority trial, or failed to prove the equivalence or the non-inferiority for equivalence and non-inferiority trials. The result of the RCT was considered significant for benefit if the *P* value was equal or lesser than 0.05 with a better outcome in the intervention group for superiority trials, or if the equivalence or non-inferiority was reached in equivalence or non-inferiority trials, or if the superiority of the intervention was reached in equivalence or non-inferiority trials. The result of the RCT was considered significant for harm if the *P* value was equal or lesser than 0.05 with a worse outcome in the intervention group for superiority trials, or if the inferiority was reached in equivalence or non-inferiority trials.

The type of intervention tested assessed whether the RCT evaluated a drug or another type of intervention [18].

### Statistical analysis

The study size was determined by the total number of RCTs published in the six journals taken into account over the studied period. The database was then split into two cohorts according to the year of publication, to control time-effect and change of policy in the journal’s editing [19]. The derivation cohort included the RCTs published on even years, and the validation cohort included the RCTs published on odd years. We described the cohorts using means, ranges, and SDs as appropriate for continuous variables and frequencies with proportion for categorical variables. We compared proportions using a *χ*^2^ test. We compared ordinal categorical variables using a Kruskal–Wallis rank sum test. There was no missing data, so no missing data imputation technique was used [20].

Logistic regression was used to identify predictive factors for publication in a high-impact general journal in the derivation cohort [19, 20]. Continuous variables were split into multiple categories according to their quartiles. A multivariate model was established to predict publication in a high-impact general journal [19, 21]. All variables were selected (regardless of their *P* value in the univariate analysis) and a stepwise procedure was used to select the final model, according to their Akaike Information Criteria (AIC) [22]. No time effect was found by entering the year of publication variable in a multivariate model. To establish a simplified score, we gave a score to each of the variables included in the final prediction model in relation to each one’s b parameter (regression coefficient) in that model [19]. The discriminative ability of the score was evaluated in both cohorts with receiver operating characteristic (ROC) curves to estimate the area under the curve (AUC), to internally validate the simplified score [21]. We used the bootstrap to internally validate the simplified score by sampling with replacement for 500 iterations [23]. The calibration of the score was graphically assessed by plotting the observed probability (Kaplan–Meier estimates) against the mean predicted probability within tenths of the predicted probabilities [21]. A probability of publication in a high-impact general journal compared to a high-impact critical care journal according to the simplified score was considered low under 20% and high over 80%. A *P* value of less than or equal to 0.05 was considered statistically significant.

We used SAS, version 9.4 (SAS Institute), for data analysis. The simplified score was developed using rigorous methodological standards, was internally validated using both split-sample and bootstrap validation, and was reported according to the Transparent Reporting of a Multivariable Prediction Model for Individual Prognosis or Diagnosis (TRIPOD) reporting guideline statement [14]. The sample size of the validation cohort met recommendations for validation studies of prediction tools, namely, a minimum of 100 events and a minimum of 100 nonevents [24].

## Results

### RCTs population

**Fig. 1:**
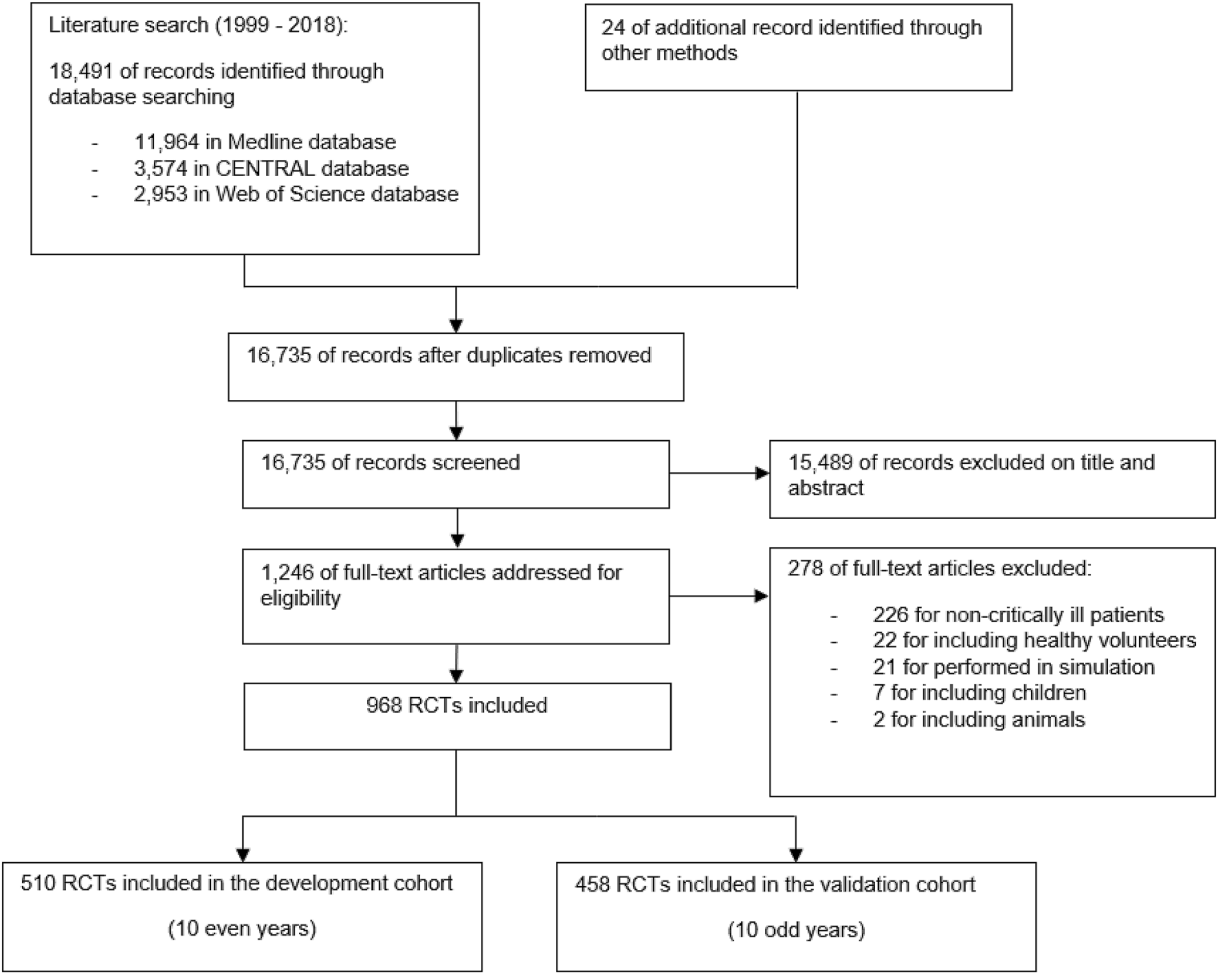
Flow chart of the study

We identified 18,515 articles using the search strategy. We excluded 1780 citations because of duplications and 15,489 citations on the initial abstract screen, because inclusion criteria were not met. After examination of the full text of the 1246 selected papers, we included 968 RCTs in this study. Figure 1 shows the study selection flowchart. 510 RCTs were included in the derivation cohort, and 458 RCTs were included in the validation cohort. 129 RCTs (25%) in the derivation cohort and 106 RCTs (23%) in the validation cohort were published in a high-impact general journal. Overall, the median sample size was 120 [interquartile range (IQR), 46 to 352], the median number of centers was 1 (IQR, 1 to 11) and the median number of countries was 1 (IQR, 1 to 1). For the analysis, the sample size was split into four categories (< 46, 46–120, 121–352, > 352) the number of centers was split into three categories (1, 2–10, > 10), and the number of countries was dichotomized (national design, international design). Characteristics of the derivation and validation cohorts are described in the electronic supplementary material (Additional file 1: Table S1).

### Final model development

Univariate and multivariate analyses in the derivation cohort are presented in Table 1. In the derivation cohort, the sample size, the number of centers involved, and mortality or composite including mortality primary outcome and topic (ventilation or miscellaneous) were independent predictive factors significantly associated with publication in a high-impact general journal.

**Table 1:** Predictive factors of publication in high-impact general journals in the derivation cohort, compared to high-impact critical care journals

Characteristic ^a^		Total (%) (n = 510)	Univariate analysis					Multivariate analysis					
			OR		95%CI	*P* value		OR	95%CI		*P* value		
Sample size	< 46	107 (21%)	1					1					
	46–120	141 (27%)	3.58		0.76–16.92	0.11		3.00	0.57–15.68		0.19		
	121–352	137 (27%)	15.35		3.58–65.77	< 0.001		8.56	1.72–42.68		0.01		
	> 352	125 (25%)	120.17		28.19–512.23	< 0.001		61.09	11.51–324.22		< 0.001		
Number of centers	1	258 (50%)	1					1					
	2–10	117 (23%)	4.74		2.55–8.79	< 0.001		1.84	0.84–4.02		0.13		
	> 10	135 (27%)	17.21		9.65–30.70	< 0.001		3.28	1.43–7.54		0.01		
International trial	Yes / No	76 (15%) / 434 (85%)	4.93		2.96–8.20	< 0.001		1.76	0.84–3.66		0.13		
Primary endpoint	Mortality alone	93 (18%)	8.66		2.26–14.24	< 0.001		3.87	1.92–7.79		0.002		
	Composite including mortality	16 (3%)	9.11		3.20–25.98	< 0.001		12.91	3.14–53.01		0.004		
	Other	401 (79%)	1					1					
Interpretation result	Significant for benefit	233 (46%)	0.42		0.27–0.64	< 0.001		1.28	0.70–2.35		0.43		
	Significant for harm	6 (1%)	1.04		0.19–5.79	0.96		0.38	0.04–3.40		0.38		
	Unsignificant	271 (53%)	1					1					
Type of intervention	Drug (Yes/No)	213 (42%) / 297 (58%)	0.71		0.48–1.06	0.10		0.80	0.43–1.49		0.48		
Country of first author	USA (Yes/No)	111 (22%) / 399 (78%)	1.34		0.84–2.13	0.53		1.31	0.69–2.48		0.41		
Topic	Sepsis	84 (17%)	1					1					
	Cardiovascular	89 (18%)	1.62		0.81–3.28	0.17		1.26	0.47–3.43		0.64		
	Ventilation	130 (25%)	1.29		0.66–2.50	0.45		6.27	2.30–17.11		< 0.001		
	Miscellaneous	207 (40%)	1.39		0.75–2.58	0.29		5.10	2.02–12.89		< 0.001		

OR: Odd Ratio, 95%CI: Confidence Interval at 95%, USA: United States of America

^a^ There was no missing data in the derivation cohort

**Fig. 2:**
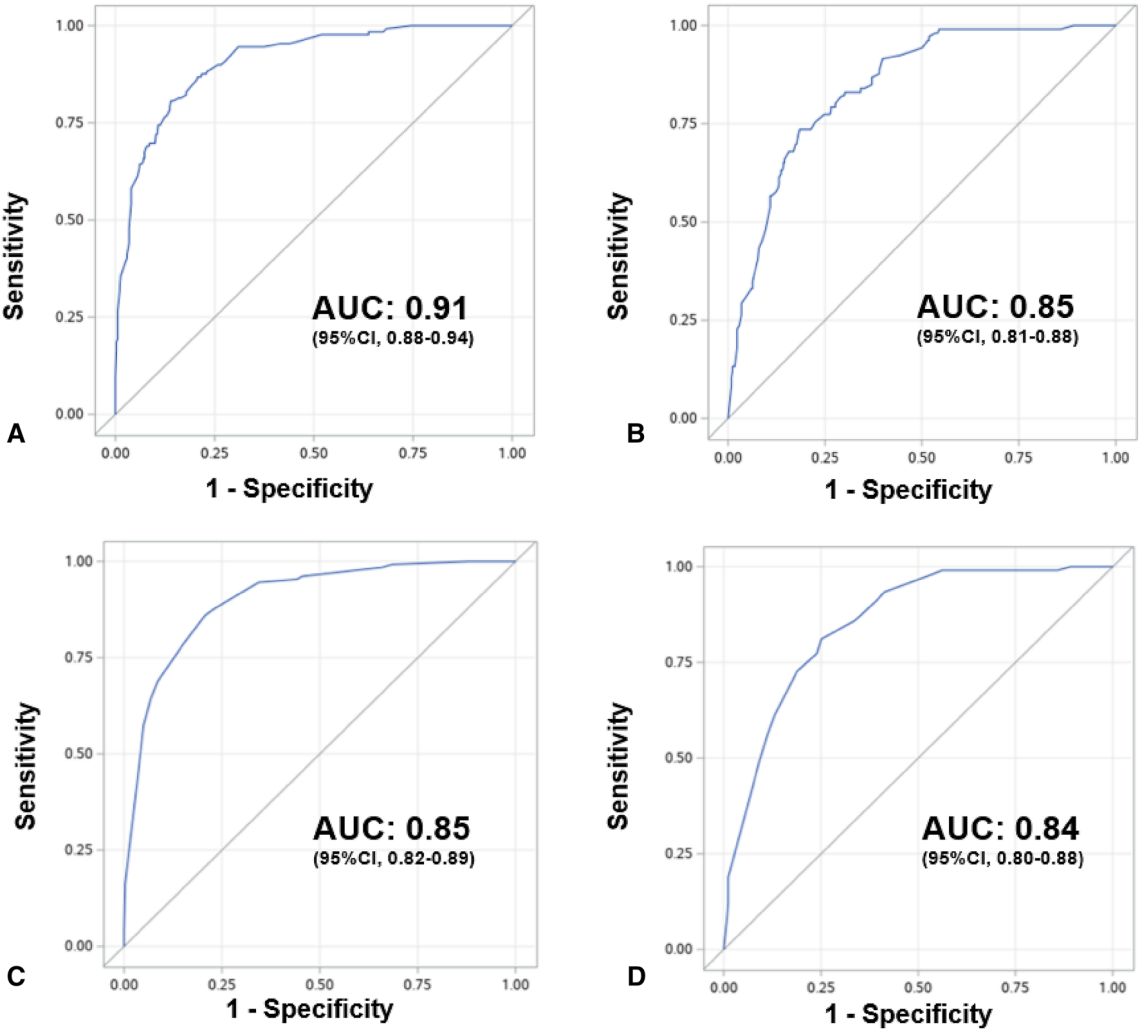
Receiver Operating Characteristic curves and Areas Under the Curve to predict publication in high-impact general journals, compared to high-impact critical care journals: **a** final model in development cohort. **b** Final model in validation cohort. **c** SCOTI score in development cohort. **d** SCOTI score in validation cohort. AUC: Area under the curve, 95%CI: Confidence Interval at 95%

The final multivariate model was constructed with the 510 RCTs of the derivation cohort and all available data. The main predictors for publication in a high-impact general journal were sample size, the number of centers involved, primary outcome, the RCT topic, and an international design. Results of the multivariate logistic regression are presented in Table 2. The AUC of the model was at 0.91 (95% Confidence Interval (95% CI), 0.88–0.94) (Fig. 2a). The calibration plot for the derivation cohort showed that the model calibration line is very close to the ideal calibration line (Additional file 1: Figure S1). There was no collinearity in the model. No time effect was found by entering the year of publication variable in a multivariate model (Additional file 1: Table S2). No significant interactions were found, neither between the outcome and the interpretation result, nor between the number of centers and the sample size (Additional file 1: Table S3).

**Table 2:** Study outcomes

Variable		*β* parameter ^a^	Odd ratio	95% CI	*P* value
Intercept		− 5.83			< 0.001
Sample size	< 46	0			
	46–120	1.13	3.09	0.60–15.99	0.18
	121–352	2.16	8.64	1.77–42.09	0.01
	> 352	4.13	62.06	11.93–322.94	< 0.001
Centers	1	0			
	2–10	0.60	1.82	0.83–3.96	0.13
	> 10	1.09	2.96	1.31–6.71	0.01
Primary endpoint	Mortality	1.28	3.60	1.81–7.15	< 0.001
	Composite including mortality	2.50	12.23	3.04–49.28	< 0.001
	Other	0			
Topic	Sepsis	0			
	Cardiovascular	0.29	1.33	0.50–3.55	0.57
	Ventilation	1.88	6.58	2.51–17.26	< 0.001
	Miscellaneous	1.69	5.40	2.17–13.44	< 0.001
International		0.53	1.70	0.82–3.53	0.15

OR: Odd Ratio, 95%CI: Confidence Interval at 95%

^a^* β* parameters: coefficients from the logistic regression model

### Final model validation

Univariate and multivariate analyses in the validation cohort are presented in the electronic supplementary material (Additional file 1: Table S4). Internal validation of the model with the validation cohort indicated high discrimination with a model AUC at 0.85 (95% CI 0.81–0.88) (Fig. 2b). The calibration plot for the validation cohort showed that the model calibration line is very close to the ideal calibration line (Additional file 1: Fig. S2).

After internal validation by bootstrap, the final model AUC was at 0.90 (95% CI 0.87–0.92).

### SCOTI score development and validation

**Fig. 3:**
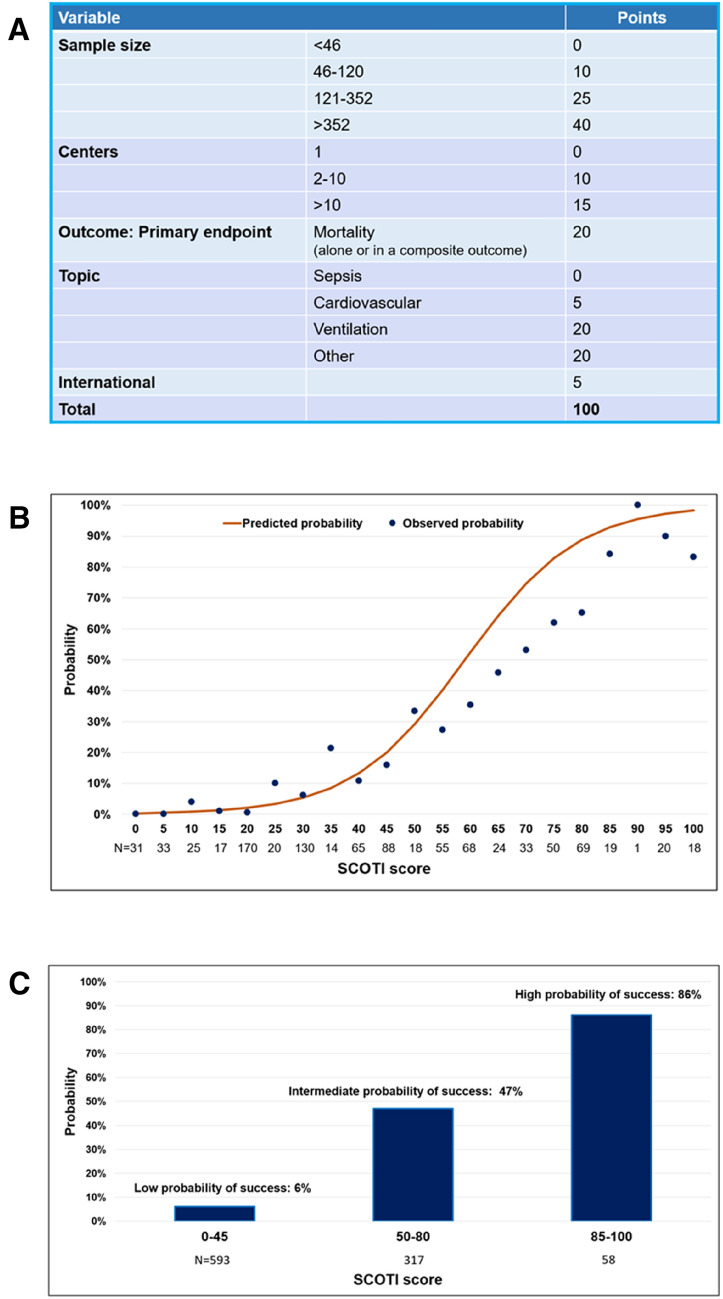
SCOTI score. **a** SCOTI score calculation worksheet. SCOTI score: sample size, centers, outcome, topic, and international score. **b** Probability of publication in high-impact general journals compared to high-impact critical care journals according to the SCOTI score: individual prediction. The plot shows predicted (orange curve) and observed (blue dots) probabilities of publication in high-impact general journals according to the SCOTI score. **c** Probability of publication in high-impact medical journals compared to high-impact critical care journals according to the SCOTI score: three categories. The plot shows observed probabilities of publication in high-impact general journals according to three groups of SCOTI score: “Low probability of success”, “Intermediate probability of success” and “High probability of success”

The simplified score (SCOTI score for Sample size, Centers, Outcome, Topic, and International score) constructed using the final model is described in Fig. 3a, ranging from 0 to 100. The model AUC was at 0.85 (95% Confidence Interval (95% CI), 0.82–0.89) (Fig. 2c). The calibration plot for the derivation cohort showed that the model calibration line is very close to the ideal calibration line (Additional file 1: Fig.7 S3). Distribution of the SCOTI score in the derivation cohort is presented in Additional file 1: Fig. S4.

Internal validation of the SCOTI score on the validation cohort indicated high discrimination with model AUC at 0.84 (95% CI 0.80–0.88) (Fig. 2d). The calibration plot for the validation cohort showed that the model calibration line is very close to the ideal calibration line (Additional file 1: Figure S5). After internal validation by bootstrap, the AUC of the simplified score was at 0.85 (95% CI 0.82–0.89). Distribution of the SCOTI score in the validation cohort is presented in Additional file 1: Figure S6.

Figure 3b presents the predicted probabilities versus the observed probability of publication in a high-impact general journal on both cohorts in function of the SCOTI score. Figure 3c presents the observed probability of publication in a high-impact general journal according to three ranges of SCOTI score. A SCOTI score between 0 and 45 results in a low probability of publication in a high-impact general journal (6%). A SCOTI score between 50 and 80 results in an intermediate probability of publication in a high-impact general journal (47%). A SCOTI score above 80 results in a high probability of publication in a high-impact general journal (86%). Additional file 1: Table S6 presents the number of RCTs published by each journal per SCOTI score category. NEJM publishes significatively less RCTs with a SCOTI score between 0 and 45 than The Lancet or The JAMA (*P* < 0.001, Kruskal–Wallis rank sum test).

A cutoff at 50 was determined by ROC analyses, to allow an excellent negative predictive value and a good positive predictive value (i.e., a probability of publication in a high-impact general journal compared to a high-impact critical care journal over 20%). In the validation cohort, positive and negative predictive values (95% CI) were 49% and 93%, respectively, with a sensitivity of 81% and a specificity of 75%.

## Discussion

In this predictive cohort of RCTs performed in the field of critical care over 20 years and published in the three highest impact general journals and the three highest impact critical care journals, sample size, number of centers, the primary endpoint, and the topic of the RCT were independent predictive factors of successful publication in a high-impact general journal in multivariate analysis, compared to a high-impact critical care journal. We designed and internally validated by split-sample and bootstrap a model and a simplified score (SCOTI score for Sample size, Centers, Outcome, Topic, and International score). This study shows that a simple easy-to-use model can predict the probability of an RCT being published in a high-impact general journal.

All of the decisive factors of the predictive model and the SCOTI score are determined at trial conception, before inclusion of the first patient [3, 4]. The result of the study does not seem to influence its chances of being published in a high-impact general journal, leading to think that there are few publication bias when it comes to well-designed RCTs [25]. This means that high-impact general journals have taken into account concerns regarding publication bias raised in the 2000s [25]. It is worth noting that it also suggests that critical care RCTs published in NEJM, The Lancet, or the JAMA are specifically designed to address the high requirements of these journals [18, 26]. High-impact general journals have already disclosed their publication policy as focusing on large, international RCTs with solid endpoints. Indeed, high-impact general journals attach importance to the methodological construction of an RCT: its sample size, its multicentric and international character, as well as an objective and pertinent outcome, assessing mortality either as primary endpoint or as a component of the primary endpoint [18]. These results are consistent with a previous study focusing on manuscripts submitted to the British Medical Journal, The Lancet and Annals of Internal Medicine, that found that manuscripts with higher methodology scores were more likely to be published in those journals [27]. In this paper either, the result of a study was not associated with its acceptance. The impact factors of the critical care journals have substantially improved during the studied period, which has probably modified the quality of the RCTs published in these journals.

It is worth noting that the RCTs included in this study were published before the COVID-19 crisis, which may have altered the topics highlighted by high-impact general journals [28]. However, in the studied period sepsis seems to be equally studied in both critical care and general journals, while ventilation trials more often attract general journals.

Our study is to our knowledge the first to highlight independent predictors of publication in a high-impact general journal in the field of critical care. Moreover, it is to our knowledge the largest database of critical care RCTs published to this date, with 968 RCTs published over 20 years in 6 high-impact journals. In this study, we were able to develop and internally validate by split sample and bootstrap a simplified score with excellent discrimination and calibration qualities (Fig. 2, Additional file 1: Figures S3, S4). We could speculate that the SCOTI score presents three major points of interest for research clinicians and journal editors. First, it could help to optimize the methodology used to design an RCT according to the selected criteria which constitute the SCOTI score (Fig. 3) when these criteria are achievable [29]. Second, the SCOTI score might help to accelerate knowledge transfer by assessing pre-submission probability of acceptance. It is an ethical issue to disclose research results to the medical community as fast as possible after trial completion. Multiple submissions to general journals of an RCT with a low probability of acceptance predicted by the SCOTI score could be limited, to avoid waste of time. Third, the SCOTI score might be useful for authors to better understand how the highest impact general journals assess critical care RCTs for possible publication. However, such a score must not be the only component of a submission process, since an RCT with a low SCOTI score but with a revolutionary concept deserves to be granted funding and wide medical diffusion. Some researchers will probably continue to submit their research to general journals rather than directly submit to critical care journals even if their SCOTI scores were low.

Notwithstanding the fact that our model was developed on critical care RCTs, its consequences might be insightful to researchers of other specialties. An external validation cohort on a different field (such as emergency medicine or cardiology) could be complementary to our analysis. Finally, although the score was built over 20 years, no effect of time was identified, neither as fixed nor random effect.

Our study also has limitations. First, we could not assess the manuscripts rejected by each journal over the period. Since our study hypothesizes that every RCT would have been published in a high-impact general journal if it had been accepted by one of them and that every RCT published in a high-impact critical care journal would have been rejected by high-impact general journals, it would have been an added value to compare both submitted and published RCTs to verify this hypothesis. Second, since there is little to no literature on this subject, variables collected and assessed for the model development were selected according to an expert panel (ADJ, GC, EA, EF, NM, SJ) and data on similar subjects [18, 30, 31]. Noteworthily, we chose to dichotomize the country of the study in United States of America versus other, while other classifications might have been used (such as English-speaking countries versus other). Moreover, we did not study in this paper the potential “human factors” (the number of publications of the first or last author, the endorsement by a large consortium or trial group, gender of authors…) influencing the publication process, since it would have highly increased the risk of collinearity with other variables [32]. Similarly, we did not focus on other methodological aspects, such as the absence of bias or the robustness of the statistical analysis. These human and methodological factors should be specifically evaluated in future works [33]. Third, we chose to include in our study the journals with the highest recent Impact Factors. From 2007 at least, data from Harhay and al. indicate that the 6 studied journals are the journals that publish the largest number of RCTs of their respective categories [18]. Likewise, we did not include in our analysis the sistership journals of the high-impact general journals (such as Lancet Respiratory Medicine), since their impact factors is closer to critical care journals than to high-impact general journals. Moreover, their existence is recent, and they cannot be evaluated from 1999 to 2018. Other methods of journals selection might have led to other journals being included. Fourth, since no linearity hypothesis could be done on any of the continuous variables, we had to categorize those variables. We split the variables into categories according to quartiles, since it is a valid and reproducible method. Fifth, we assessed high-impact general journals as a whole, but they might have different policies from one another. Sixth, comparing the characteristics of the trials accepted in the 6 included journals to journals with lower Impact Factors might have been very interesting, since publishing a paper in one of the 3 highest Impact Factor critical care journals is becoming more and more challenging.

## Conclusions

In this predictive cohort of critical care RCTs, the sample size, the number of centers, the primary endpoint, and the topic of an RCT were independent predictive factors of successful publication in a high-impact general journal in multivariate analysis, compared to a high-impact critical care journal. The SCOTI score was developed and internally validated by split sample and bootstrap. It predicts the probability of a critical care RCT being published in a high-impact general journal.

## Supplementary Information

**Additional file 1. Appendix S1**. Search strategy. **Table S1.** Description of derivation and validation cohorts. **Table S2.** Multivariate model to assess time effect. **Table S3.** Multivariate model to assess interaction between outcome and interpretation result and interaction between number of centers and sample size. **Table S4.** Univariate and multivariate analysis on the development cohort. **Table S5.** Univariate and multivariate analysis on the validation cohort. **Table S6.** Number of RCTs per SCOTI score category per journal. **Figure S1.** Calibration plot for the final model on the derivation cohort. **Figure S2.** Calibration plot for the final model on the validation cohort. **Figure S3.** Calibration plot for the SCOTI score on the derivation cohort. **Figure S4.** Distribution of the SCOTI score in the derivation cohort. **Figure S5.** Calibration plot for the SCOTI score on the validation cohort. **Figure S6.** Distribution of the SCOTI score in the validation cohort.

## Data Availability

Research data and other material (e.g., study protocol and statistical analysis plan) will be made available to the scientific community, immediately on publication, with as few restrictions as possible. All requests should be submitted to the corresponding author who will review with the other investigators for consideration. A data use agreement will be required before the release of participant data and institutional review board approval as appropriate.

## Abbreviations

RCTs: Randomized controlled trials
ICM: Intensive Care Medicine
AJRCCM: The American Journal of Respiratory and Critical Care Medicine
CCM: Critical Care Medicine
NEJM: New England Journal of Medicine
JAMA: Journal of the American Medical Association
AIC: Akaike Information Criteria
ROC: Receiver operating characteristic
AUC: Area under the curve
SCOTI: Sample size, Centers, Outcome, Topic, and International score
