# Supplementary material for: A multivariate model for successful publication of intensive care medicine randomized controlled trials in the highest impact factor journals: the SCOTI score
Source: Ann Intensive Care. 2021 Nov 27;11:165. doi: 10.1186/s13613-021-00954-x (PMC8626742; doi:10.1186/s13613-021-00954-x)
Supplement: Supplementary file 1 — Additional file 1. Appendix S1. Search strategy. Table S1. Description of derivation and validation cohorts. Table S2. Multivariate model to assess time effect. Table S3. Multivariate model to assess interaction between outcome and interpretation result and interaction between number of centers and sample size. Table S4. Univariate and multivariate analysis on the development cohort. Table S5. Univariate and multivariate analysis on the validation cohort. Table S6. Number of RCTs per SCOTI score category per journal. Figure S1. Calibration plot for the final model on the derivation cohort. Figure S2. Calibration plot for the final model on the validation cohort. Figure S3. Calibration plot for the SCOTI score on the derivation cohort. Figure S4. Distribution of the SCOTI score in the derivation cohort. Figure S5. Calibration plot for the SCOTI score on the validation cohort. Figure S6. Distribution of the SCOTI score in the validation cohort. [file 13613_2021_954_MOESM1_ESM.docx]

**ADDITIONAL MATERIAL - SUMMARY**

**Appendix S1. Search strategy**

**Table S1. Description of derivation and validation cohorts**

**Table S2. Multivariate model to assess time effect**

**Table S3. Multivariate model to assess interaction between outcome and interpretation result and interaction between number of centers and sample size**

**Table S4. Univariate and multivariate analysis on the development cohort**

**Table S5. Univariate and multivariate analysis on the validation cohort**

**Table S6. Number of RCTs per SCOTI score category per journal**

**Figure S 1. Calibration plot for the final model on the derivation cohort**

**Figure S 2. Calibration plot for the final model on the validation cohort**

**Figure S 3. Calibration plot for the SCOTI score on the derivation cohort**

**Figure S 4. Distribution of the SCOTI score in the derivation cohort**

**Figure S 5. Calibration plot for the SCOTI score on the validation cohort**

**Figure S 6. Distribution of the SCOTI score in the validation cohort**

**Appendix S1. Search strategy**

**Medline**

| **#** | **Searches** | **Results** |
| --- | --- | --- |
| 1 | 1999/01/01:2019/01/01[Date - MeSH] AND "JAMA"[Journal] AND ("clinical trials as topic"[MeSH Terms] OR ("clinical"[All Fields] AND "trials"[All Fields] AND "topic"[All Fields]) OR "clinical trials as topic"[All Fields] OR "trial"[All Fields] OR "trial s"[All Fields] OR "trialed"[All Fields] OR "trialing"[All Fields] OR "trials"[All Fields] OR ("random allocation"[MeSH Terms] OR ("random"[All Fields] AND "allocation"[All Fields]) OR "random allocation"[All Fields] OR "random"[All Fields] OR "randomization"[All Fields] OR "randomized"[All Fields] OR "randomisation"[All Fields] OR "randomisations"[All Fields] OR "randomise"[All Fields] OR "randomised"[All Fields] OR "randomising"[All Fields] OR "randomizations"[All Fields] OR "randomize"[All Fields] OR "randomizes"[All Fields] OR "randomizing"[All Fields] OR "randomness"[All Fields] OR "randoms"[All Fields])) | 3504 |
| 2 | 1999/01/01:2019/01/01[Date - MeSH] AND "Lancet"[Journal] AND ("clinical trials as topic"[MeSH Terms] OR ("clinical"[All Fields] AND "trials"[All Fields] AND "topic"[All Fields]) OR "clinical trials as topic"[All Fields] OR "trial"[All Fields] OR "trial s"[All Fields] OR "trialed"[All Fields] OR "trialing"[All Fields] OR "trials"[All Fields] OR ("random allocation"[MeSH Terms] OR ("random"[All Fields] AND "allocation"[All Fields]) OR "random allocation"[All Fields] OR "random"[All Fields] OR "randomization"[All Fields] OR "randomized"[All Fields] OR "randomisation"[All Fields] OR "randomisations"[All Fields] OR "randomise"[All Fields] OR "randomised"[All Fields] OR "randomising"[All Fields] OR "randomizations"[All Fields] OR "randomize"[All Fields] OR "randomizes"[All Fields] OR "randomizing"[All Fields] OR "randomness"[All Fields] OR "randoms"[All Fields])) | 4968 |
| 3 | 1999/01/01:2019/01/01[Date - MeSH] AND "N Engl J Med"[Journal] AND ("clinical trials as topic"[MeSH Terms] OR ("clinical"[All Fields] AND "trials"[All Fields] AND "topic"[All Fields]) OR "clinical trials as topic"[All Fields] OR "trial"[All Fields] OR "trial s"[All Fields] OR "trialed"[All Fields] OR "trialing"[All Fields] OR "trials"[All Fields] OR ("random allocation"[MeSH Terms] OR ("random"[All Fields] AND "allocation"[All Fields]) OR "random allocation"[All Fields] OR "random"[All Fields] OR "randomization"[All Fields] OR "randomized"[All Fields] OR "randomisation"[All Fields] OR "randomisations"[All Fields] OR "randomise"[All Fields] OR "randomised"[All Fields] OR "randomising"[All Fields] OR "randomizations"[All Fields] OR "randomize"[All Fields] OR "randomizes"[All Fields] OR "randomizing"[All Fields] OR "randomness"[All Fields] OR "randoms"[All Fields])) | 3552 |
| 4 | 1 OR 2 OR 3 | 11964 |

**CENTRAL**

| **#** | **Searches** | **Results** |
| --- | --- | --- |
| 1 | ("critical care") | 20186 |
| 2 | ("intensive care") | 32674 |
| 3 | ("emergency") OR ("intensive") OR ("critical") | 111540 |
| 4 | ("randomised") | 1125847 |
| 5 | ("controlled clinical trial"):ti,ab,kw | 166942 |
| 6 | (JAMA) OR (Journal of the American Medical Association) | 15231 |
| 7 | (NEJM) OR (N Eng J Med) OR (New England Journal of Medicine) | 7359 |
| 8 | (Lancet) OR (The Lancet) | 14407 |
| 9 | #1 OR #2 OR #3 | 107930 |
| 10 | #4 OR #5 | 1184361 |
| 11 | #6 OR #7 OR #8 | 36648 |
| 12 | #9 AND #10 AND #11 | 3574 |

**Web of Science**

| **#** | **Searches** | **Results** |
| --- | --- | --- |
| 1 | AB=("critical" OR "critically") | 1659264 |
| 2 | TI="care" | 552918 |
| 3 | AB="intensive" | 348017 |
| 4 | #3 OR #2 OR #1 | 2435703 |
| 5 | SO= (NEW ENGLAND JOURNAL OF MEDICINE OR JOURNAL OF THE AMERICAN MEDICAL ASSOCIATION OR LANCET) | 411938 |
| 6 | #5 AND #4 | 7124 |
| 7 | PY=1999-2018 | 43852097 |
| 8 | #6 AND #7 | 2953 |

**Table S1. Description of derivation and validation cohorts**

| **Characteristic** ^a^ | | **Derivation cohort (%)**  **(n=510)** | **Validation cohort (%)**  **(n=458)** | **Total (%)**  **(n=968)** |
| --- | --- | --- | --- | --- |
| **Journal** |  |  |  |  |
| ***General journals*** |  | 129 (25%) | 106 (23%) | 235 (24%) |
|  | NEJM | 48 (9%) | 37 (8%) | 85 (8%) |
|  | The Lancet | 18 (4%) | 20 (4%) | 38 (4%) |
|  | JAMA | 63 (12%) | 59 (13%) | 112 (12%) |
| ***Critical care journals*** |  | 381 (75%) | 352 (77%) | 733 (76%) |
|  | ICM | 135 (26%) | 129 (28%) | 264 (27%) |
|  | AJRCCM | 43 (8%) | 44 (10%) | 87 (9%) |
|  | CCM | 203 (40%) | 179 (39%) | 382 (40%) |
| **Sample size** | <46 | 107 (21%) | 134 (29%) | 241 (25%) |
|  | 46-120 | 141 (27%) | 96 (22%) | 237 (24%) |
|  | 121-352 | 137 (27%) | 111 (24%) | 248 (26%) |
|  | >352 | 125 (25%) | 117 (25%) | 242 (25%) |
| **Number of centers** | 1 | 258 (50%) | 253 (55%) | 511 (52%) |
|  | 2-10 | 117 (23%) | 94 (21%) | 211 (23%) |
|  | >10 | 135 (27%) | 111 (24%) | 246 (25%) |
| **International trial** | Yes / No | 76 (15%) / 434 (85%) | 66 (14%) / 392 (86%) | 142 (15%) / 826 (85%) |
| **Primary endpoint** | Mortality alone | 93 (18%) | 75 (17%) | 168 (17%) |
|  | Composite including mortality | 16 (3%) | 11 (2%) | 27 (3%) |
|  | Other | 401 (79%) | 372 (81%) | 773 (80%) |
| **Interpretation result** | Significant for benefit | 233 (46%) | 226 (49%) | 459 (47%) |
|  | Significant for harm | 6 (1%) | 14 (3%) | 20 (2%) |
|  | Unsignificant | 271 (53%) | 218 (48%) | 489 (51%) |
| **Type of intervention** | Drug (Yes/No) | 213 (42%) / 297 (58%) | 208 (45%) / 258 (55%) | 421 (43%) / 547 (57%) |
| **Country of first author** | USA (Yes/No) | 111 (22%) / 399 (78%) | 84 (18%) / 374 (82%) | 195 (20%) / 773 (80%) |
| **Topic** | Sepsis | 84 (17%) | 96 (21%) | 180 (18%) |
|  | Cardiovascular | 89 (18%) | 70 (15%) | 159 (17%) |
|  | Ventilation | 130 (25%) | 120 (26%) | 250 (26%) |
|  | Miscellaneous | 207 (40%) | 172 (38%) | 379 (39%) |

Abbreviations: NEJM: New England Journal of Medicine, JAMA: Journal of the American Medical Association, ICM: Intensive Care Medicine, AJRCCM: American Journal of Respiratory and Critical Care Medicine, CCM: Critical Care Medicine, USA: United States of America.

^a^ There was no missing data in both cohorts

**Table S2. Multivariate model to assess time effect**

| **Variable** | | **β parameter** ^a^ | **Standard error** | ***P* value** |
| --- | --- | --- | --- | --- |
| Intercept |  | -3.29 | 0.8969 | <0.001 |
| Sample size | <46 | 0 |  |  |
|  | 46-120 | 1.04 | 0.86 | 0.23 |
|  | 121-352 | 1.90 | 0.84 | 0.02 |
|  | >352 | 4.02 | 0.88 | <0.001 |
| Centers | 1 | 0 |  |  |
|  | 2-10 | 0.31 | 0.43 | 0.14 |
|  | >10 | 1.16 | 0.44 | 0.01 |
| Primary endpoint | Mortality | 1.62 | 0.39 | <0.001 |
|  | Composite including mortality | 2.57 | 0.74 | <0.001 |
|  | Other | 0 |  |  |
| Topic | Sepsis | 0 |  |  |
|  | Cardiovascular | 0.28 | 0.52 | 0.63 |
|  | Ventilation | 2.02 | 0.41 | <0.001 |
|  | Miscellaneous | 1.26 | 0.53 | <0.001 |
| International |  | 0.58 | 0.39 | 0.14 |
| Year |  | 0.19 | 0.68 | 0.77 |

^a^ β parameters: coefficients from the logistic regression model.

**Table S3. Multivariate model to assess interaction between outcome and interpretation result and interaction between number of centers and sample size**

| **Variable** | | **β parameter** ^a^ | **Standard error** | ***P* value** |
| --- | --- | --- | --- | --- |
| Intercept |  | -4.60 | 1.10 | <0.001 |
| Sample size | <46 | 0 |  |  |
|  | 46-120 | 1.79 | 1.53 | 0.13 |
|  | 121-352 | 3.15 | 1.12 | 0.01 |
|  | >352 | 4.47 | 1.25 | <0.001 |
| Centers | 1 | 0 |  |  |
|  | 2-10 | 2.77 | 2.03 | 0.17 |
|  | >10 | 1.61 | 0.77 | 0.03 |
| Primary endpoint | Mortality | 1.21 | 0.43 | 0.01 |
|  | Composite including mortality | 3.25 | 0.92 | <0.001 |
|  | Other | 0 |  |  |
| Topic | Sepsis | 0 |  |  |
|  | Cardiovascular | 0.37 | 0.50 | 0.68 |
|  | Ventilation | 2.12 | 0.41 | <0.001 |
|  | Miscellaneous | 1.12 | 0.52 | 0.01 |
| International |  | 0.56 | 0.39 | 0.15 |
| Outcome * Interpretation result |  |  |  |  |
|  | Mortality*Significant for benefit | 1.12 | 0.82 | 0.17 |
|  | Composite*Significant for benefit | -2.71 | 1.67 | 0.10 |
|  | Mortality*Significant for harm | 13.18 | 748.99 | 0.99 |
|  | Composite*Significant for harm | 26.04 | 1636.82 | 0.99 |
| Sample size * Centers |  |  |  |  |
|  | 2-10 centers * 46-120 patients | -2.91 | 2.22 | 0.19 |
|  | >10 centers * 46-120 patients | 0.29 | 1.15 | 0.80 |
|  | 2-10 centers * 121-352 patients | -2.58 | 2.11 | 0.22 |
|  | >10 centers * 121-352 patients | -0.82 | 0.94 | 0.38 |
|  | 2-10 centers * >352 patients | -1.69 | 2.19 | 0.44 |
|  | >10 centers * >352 patients | -2.76 | 1.67 | 0.10 |
|  |  |  |  |  |

^a^ β parameters: coefficients from the logistic regression model.

**Table S4. Univariate and multivariate analysis on the development cohort** (n=510)

| **Characteristic** ^a^ | | **General journals (%)**  **(n=129)** |  | **Critical care journals (%)**  **(n=381)** | **Univariate analysis** | | | **Multivariate analysis** | | |
| --- | --- | --- | --- | --- | --- | --- | --- | --- | --- | --- |
|  | |  |  |  | **OR** | **95% CI** | **P value** | **OR** | **95%CI** | **Pvalue** |
| **Sample size** | <46 | 2 (2%) |  | 105 (27%) | 1 |  |  | 1 |  |  |
|  | 46-120 | 9 (7%) |  | 132 (35%) | 3.58 | 0.76-16.92 | 0.11 | 3.00 | 0.57-15.68 | 0.19 |
|  | 121-352 | 31 (24%) |  | 106 (28%) | 15.35 | 3.58-65.77 | <0.001 | 8.56 | 1.72-42.68 | 0.01 |
|  | >352 | 87 (67%) |  | 38 (10%) | 120.17 | 28.19-512-23 | <0.001 | 61.09 | 11.51-324.22 | <0.001 |
| **Number of centers** | 1 | 19 (15%) |  | 239 (63%) | 1 |  |  | 1 |  |  |
|  | 2-10 | 32 (25%) |  | 85 (22%) | 4.74 | 2.55-8.79 | <0.001 | 1.84 | 0.84-4.02 | 0.13 |
|  | >10 | 78 (60%) |  | 57 (15%) | 17.21 | 9.65-30.70 | <0.001 | 3.28 | 1.43-7.54 | 0.01 |
| **International trial** | Yes / No | 42 (33%) / 87 (67%) |  | 34 (9%) / 347 (91%) | 4.93 | 2.96-8.20 | <0.001 | 1.76 | 0.84-3.66 | 0.13 |
| **Primary endpoint** | Mortality alone | 57 (44%) |  | 36 (9%) | 8.66 | 2.26-14.24 | <0.001 | 3.87 | 1.92-7.79 | 0.002 |
|  | Composite including mortality | 10 (8%) |  | 6 (2%) | 9.11 | 3.20-25.98 | <0.001 | 12.91 | 3.14-53.01 | 0.004 |
|  | Other | 62 (48%) |  | 339 (89%) | 1 |  |  | 1 |  |  |
| **Interpretation result** | Significant for benefit | 39 (30%) |  | 194 (51%) | 0.42 | 0.27-0.64 | <0.001 | 1.28 | 0.70-2.35 | 0.43 |
|  | Significant for harm | 2 (2%) |  | 4 (1%) | 1.04 | 0.19-5.79 | 0.96 | 0.38 | 0.04-3.40 | 0.38 |
|  | Unsignificant | 88 (68%) |  | 183 (48%) | 1 |  |  | 1 |  |  |
| **Type of intervention** | Drug (Yes/No) | 46 (36%) / 83 (64%) |  | 167 (44%) / 214 (56%) | 0.71 | 0.48-1.06 | 0.10 | 0.80 | 0.43-1.49 | 0.48 |
| **Country of first author** | USA (Yes/No) | 33 (26%) / 96 (74%) |  | 78 (20%) / 303 (80%) | 1.34 | 0.84-2.13 | 0.53 | 1.31 | 0.69-2.48 | 0.41 |
| **Topic** | Sepsis | 17 (13%) |  | 67 (18%) | 1 |  |  | 1 |  |  |
|  | Cardiovascular | 26 (20%) |  | 63 (17%) | 1.62 | 0.81-3.28 | 0.17 | 1.26 | 0.47-3.43 | 0.64 |
|  | Ventilation | 33 (26%) |  | 97 (25%) | 1.29 | 0.66-2.50 | 0.45 | 6.27 | 2.30-17.11 | <0.001 |
|  | Miscellaneous | 53 (41%) |  | 154 (40%) | 1.39 | 0.75-2.58 | 0.29 | 5.10 | 2.02-12.89 | <0.001 |

Abbreviations: OR: Odd Ratio, 95%CI: Confidence Interval at 95%, USA: United States of America.

^a^ There was no missing data in both cohorts

**Table S5. Univariate and multivariate analysis on the validation cohort** (n=458)

| **Characteristic** ^a^ | | **General journals (%)**  **(n=106)** |  | **Critical care journals (%)**  **(n=352)** | **Univariate analysis** | | | **Multivariate analysis** | | |
| --- | --- | --- | --- | --- | --- | --- | --- | --- | --- | --- |
|  | |  |  |  | **OR** | **95% CI** | **P value** | **OR** | **95%CI** | **Pvalue** |
| **Sample size** | <46 | 1 (1%) |  | 133 (38%) | 1 |  |  | 1 |  |  |
|  | 46-120 | 10 (9%) |  | 86 (24%) | 15.46 | 1.95-122.96 | 0.001 | 8.83 | 1.06-74.00 | 0.04 |
|  | 121-352 | 30 (28%) |  | 81 (23%) | 49.25 | 6.59-368.09 | <0.001 | 15.91 | 1.95-129.48 | 0.01 |
|  | >352 | 65 (62%) |  | 52 (15%) | 166.23 | 22.48 - >999.99 | <0.001 | 30.58 | 3.57-262.27 | 0.002 |
| **Number of centers** | 1 | 17 (16%) |  | 236 (67%) | 1 |  |  | 1 |  |  |
|  | 2-10 | 30 (28%) |  | 64 (18%) | 6.51 | 3.38-12.54 | <0.001 | 3.65 | 1.63-8.18 | 0.002 |
|  | >10 | 59 (56%) |  | 52 (15%) | 15.75 | 8.50-29.21 | <0.001 | 5.88 | 2.27-15.26 | <0.001 |
| **International trial** | Yes / No | 32 (30%) / 74 (70%) |  | 34 (10%) / 318 (90%) | 4.05 | 2.35-6.98 | <0.001 | 1.05 | 0.46-2.41 | 0.90 |
| **Primary endpoint** | Mortality alone | 45 (42%) |  | 30 (9%) | 8.29 | 4.82-14.24 | <0.001 | 4.70 | 2.15-10.25 | <0.001 |
|  | Composite including mortality | 4 (4%) |  | 7 (2%) | 3.16 | 0.89-11.14 | 0.07 | 5.36 | 1.03-27.84 | 0.05 |
|  | Other | 57 (54%) |  | 315 (89%) | 1 |  |  | 1 |  |  |
| **Interpretation result** | Significant for benefit | 46 (43%) |  | 180 (51%) | 0.82 | 0.52-1.28 | 0.37 | 3.27 | 1.63-2.35 | 0.001 |
|  | Significant for harm | 8 (8%) |  | 6 (2%) | 4.26 | 1.41-12.83 | 0.01 | 4.33 | 1.08-17.41 | 0.04 |
|  | Unsignificant | 52 (49%) |  | 166 (47%) | 1 |  |  | 1 |  |  |
| **Type of intervention** | Drug (Yes/No) | 39 (37%) / 67 (63%) |  | 169 (48%) / 183 (52%) | 0.71 | 0.47-1.07 | 0.10 | 0.71 | 0.37-1.37 | 0.30 |
| **Country of first author** | USA (Yes/No) | 30 (28%) / 76 (72%) |  | 54 (15%) / 298 (85%) | 2.18 | 1.31-3.64 | 0.002 | 1.68 | 0.86-3.25 | 0.13 |
| **Topic** | Sepsis | 19 (18%) |  | 77 (22%) | 1 |  |  | 1 |  |  |
|  | Cardiovascular | 24 (22%) |  | 46 (13%) | 2.11 | 1.05-4.28 | 0.04 | 4.06 | 1.51-10.94 | 0.006 |
|  | Ventilation | 21 (20%) |  | 99 (28%) | 0.86 | 0.43-1.71 | 0.67 | 2.30 | 0.88-6.01 | 0.09 |
|  | Miscellaneous | 42 (40%) |  | 130 (37%) | 0.62 | 0.27-1.39 | 0.25 | 1.68 | 0.57-4.92 | 0.34 |

Abbreviations: OR: Odd Ratio, 95%CI: Confidence Interval at 95%, USA: United States of America.

^a^ There was no missing data in both cohorts

**Table S6. Number of RCTs per SCOTI score category per journal**

| **Journal** | | **Low probabilty**  **SCOTI [0-45]** | **Intermediate probability**  **SCOTI [50-80]** | **High probability**  **SCOTI [85-100]** |
| --- | --- | --- | --- | --- |
| ***General journals*** |  | 43 (18%) | 147 (63%) | 45 (19%) |
|  | NEJM | 9 (11%) | 51 (60%) | 25 (29%) |
|  | The Lancet | 8 (21%) | 25 (66%) | 5 (13%) |
|  | JAMA | 26 (23%) | 71 (64%) | 15 (13%) |
| ***Critical care journals*** |  | 580 (79%) | 46 (20%) | 4 (1%) |
|  | ICM | 214 (81%) | 46 (17%) | 4 (2%) |
|  | AJRCCM | 53 (61%) | 32 (37%) | 2 (2%) |
|  | CCM | 313 (81%) | 68 (18%) | 1 (1%) |

Abbreviations: NEJM: New England Journal of Medicine, JAMA: Journal of the American Medical Association, ICM: Intensive Care Medicine, AJRCCM: American Journal of Respiratory and Critical Care Medicine, CCM: Critical Care Medicine

**Figure S 1. Calibration plot for the final model on the derivation cohort**

**
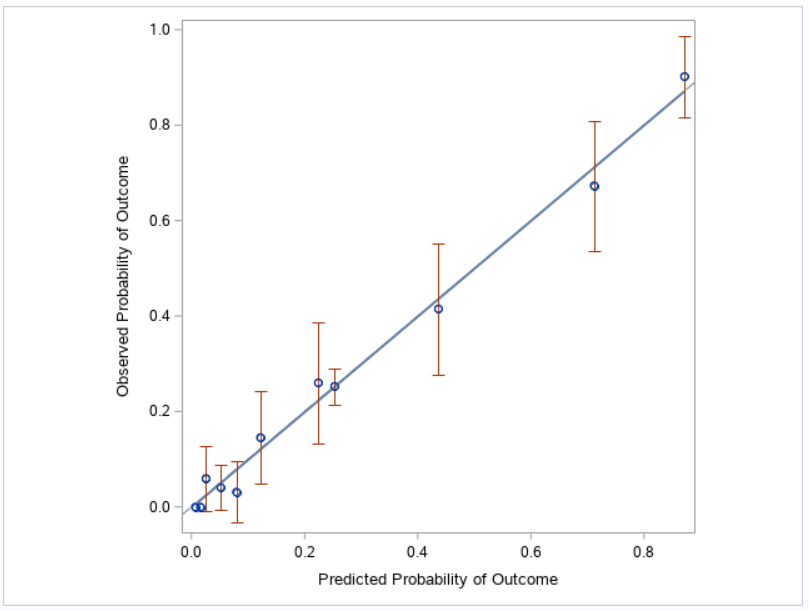
**

**Figure S 2. Calibration plot for the final model on the validation cohort**


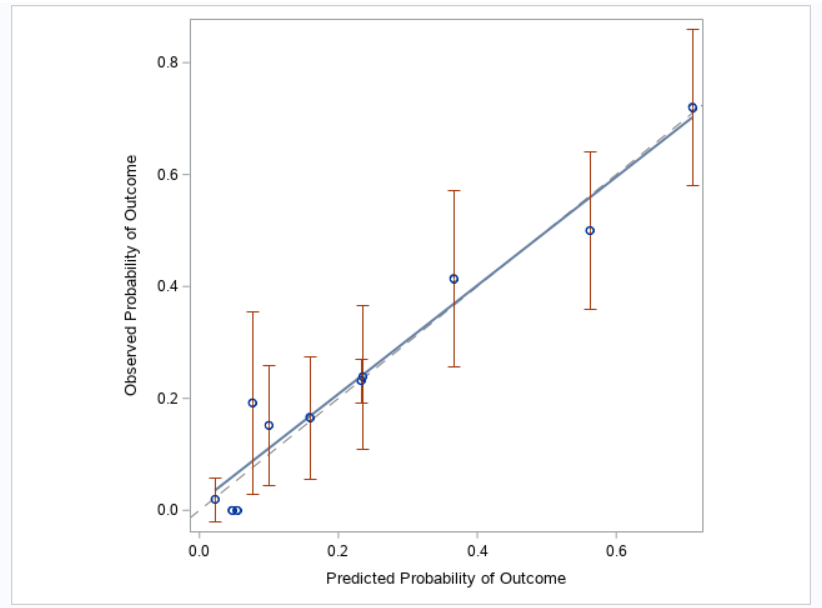


**Figure S 3. Calibration plot for the SCOTI score on the derivation cohort**


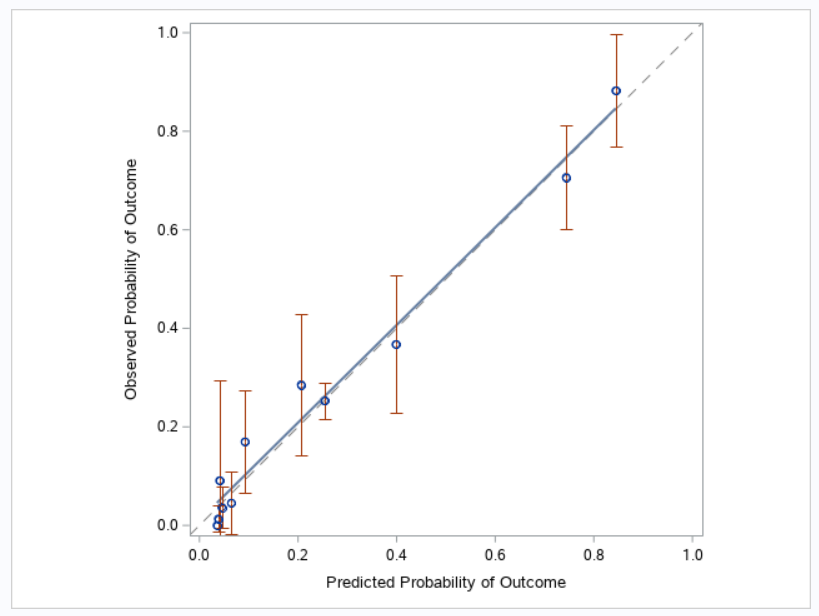


**Figure S 4. Distribution of the SCOTI score in the derivation cohort**


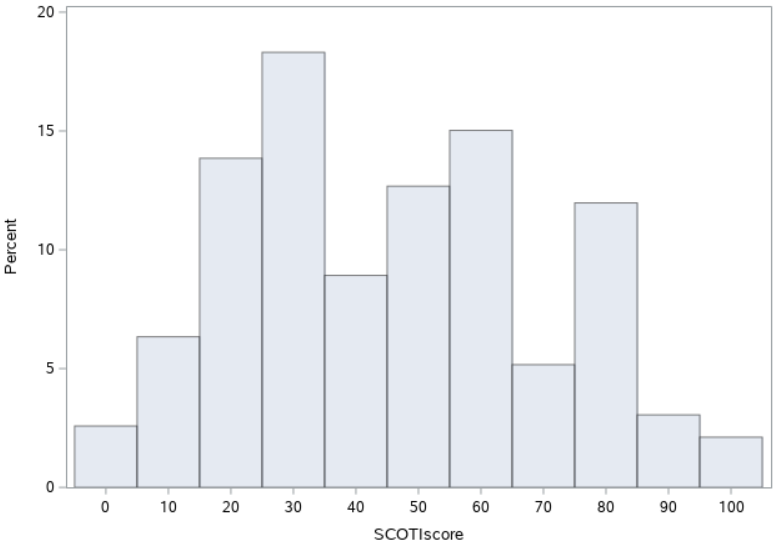


**Figure S 5. Calibration plot for the SCOTI score on the validation cohort**


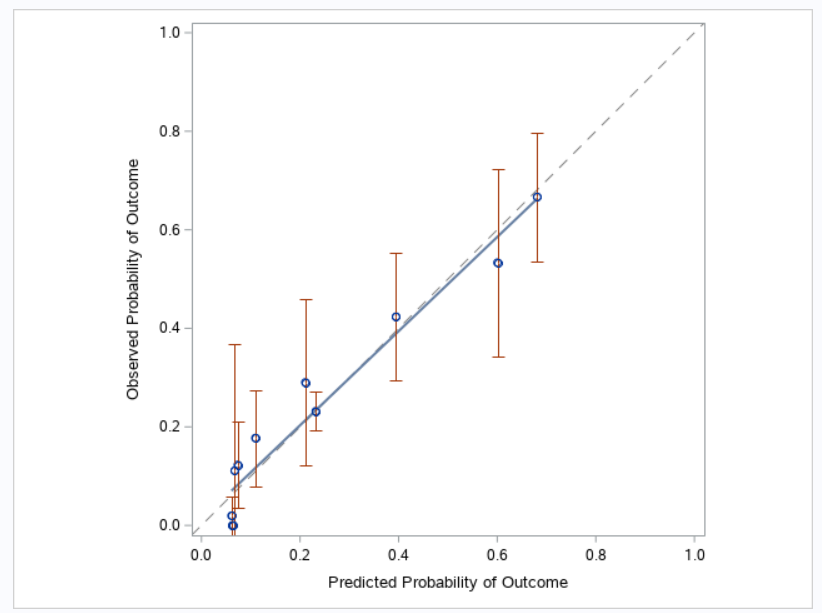


**Figure S 6. Distribution of the SCOTI score in the validation cohort**

**
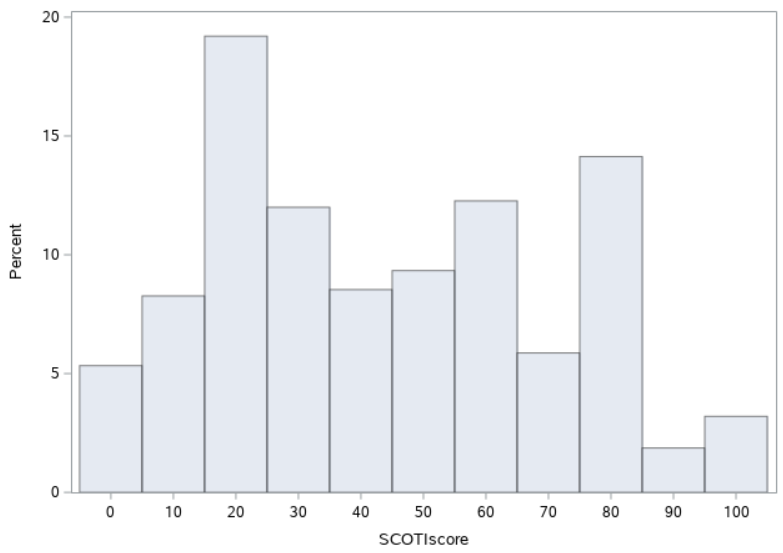
**
